# Spiking neural network model of reinforcement learning in the honeybee implemented on the GPU

**DOI:** 10.1186/1471-2202-16-S1-P181

**Published:** 2015-12-18

**Authors:** Esin Yavuz, Pascale Maul, Thomas Nowotny

**Affiliations:** 1 CCNR, School of Engineering and Informatics, University of Sussex, Falmer, Brighton, UK; 2 Institute of Cognitive Science, University of Osnabrück, 49069 Osnabrück, Germany

## 

Honeybees can learn and perform complex behavioral tasks despite their small brains that contain less than a million neurons. At the same time they are accessible to physiological experiments and the relatively small number of neurons in their brain lends itself to quite detailed numerical simulations. Bees therefore are a good model system for studying sensory cognition and reinforcement learning.

**Figure 1:**
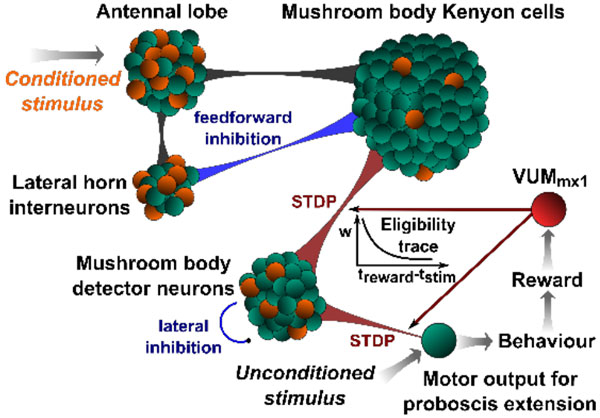
**Network diagram for the hypothesized model of reinforcement learning in the honeybee olfactory system**. Excitatory connections are shown in black, inhibitory connections in blue and learning synapses in red. Grey arrows represent the abstractions modeled by implicit mechanisms. The model consists of the antennal lobe, lateral horn interneurons, mushroom body Kenyon cells and lobe neurons, and an octopaminergic/dopaminergic pathway for reinforcement, classically considered to be the VUM_mx1 _neuron. A conditioned stimulus is paired with an unconditioned stimulus (sugar to the antenna) to elicit the behavioral response (proboscis extension) in the training phase, which can then be rewarded by letting the bees drink. The association with reward facilitates plasticity in the synapses between the Kenyon cells and lobe neurons and between lobe neurons and pre-motor neurons. The size of weight changes is determined by an eligibility trace as a function of the delay between stimulus and reward.

We have shown in earlier work [1] that the anatomy and known electrophysiological properties of the olfactory pathway of insects in combination with spike-timing dependent plasticity (STDP) and lateral inhibition lend themselves to an unsupervised self-organization of synaptic connections for the recognition of odors. Here we extend this model by adding mechanisms of reinforcement learning, as suggested by [2] (see Figure 1). We employ a three factor learning rule where plasticity is governed by pre-synaptic and post-synaptic activity and a global octopaminergic/dopaminergic reinforcement signal, triggered by a reward. We investigated the role of feed-forward and feedback mechanisms, as well as the role of the connectivity initially achieved by unsupervised STDP.

Our model is implemented in the GeNN [3] framework, which facilitates the use of GPUs for spiking neural network simulations using a code generation framework. Because of the massive parallelism provided by GPUs, we can simulate tens of thousands of neurons in real time in the sparse firing regime relevant here. We investigated optimization strategies and neuron and synapse model choices for a better performance on the GPU. The model presented here is a stepping-stone to more sophisticated learning models and multi-sensory integration in the Green Brain Project [4], in which we aim to control a flying robot with a simulation of learning and decision making mechanisms in the honeybee related both to the olfactory and visual pathways.
